# Supplementary material for: Trial Assay for Safe First-Aid Protocol for the Stinging Sea Anemone Anemonia viridis (Cnidaria: Anthozoa) and a Severe Toxic Reaction
Source: Toxins (Basel). 2022 Jan 1;14(1):27. doi: 10.3390/toxins14010027 (PMC8780947; doi:10.3390/toxins14010027)
Supplement: Supplementary file 1 [file toxins-14-00027-s001.zip › toxins-1520464-supplementary.pdf]

Guía de identificación de MEDUSAS Y OTROS ORGANISMOS GELATINOSOS

LA MAR DE MEDUSAS

Con la colaboración de

Institut de Ciències del Mar

CSIC
Consejo Superior de Investigaciones Científicas

Gobierno de España
Ministerio de Ciencia e Innovación

FECYT
Fundación Española para la Ciencia y la Tecnología

¿Has visto una medusa?
¿Quieres participar en un proyecto de Ciencia Ciudadana?
Puedes enviar tus avistamientos a

Observadores del Mar

Proyecto Alerta Medusas

¿Cómo funciona?

Haz una foto → ***Súbela** a la web* → ***Dialoga** y **obtén validación** con los equipos científicos* → *Amplía el **conocimiento***

www.observadoresdelmar.es

@obsdelmar

Clasificación taxonómica

Las medusas son invertebrados marinos del zooplancton gelatinoso que pertenecen a tres filos diferentes: Chordata, Ctenophora y Cnidaria.

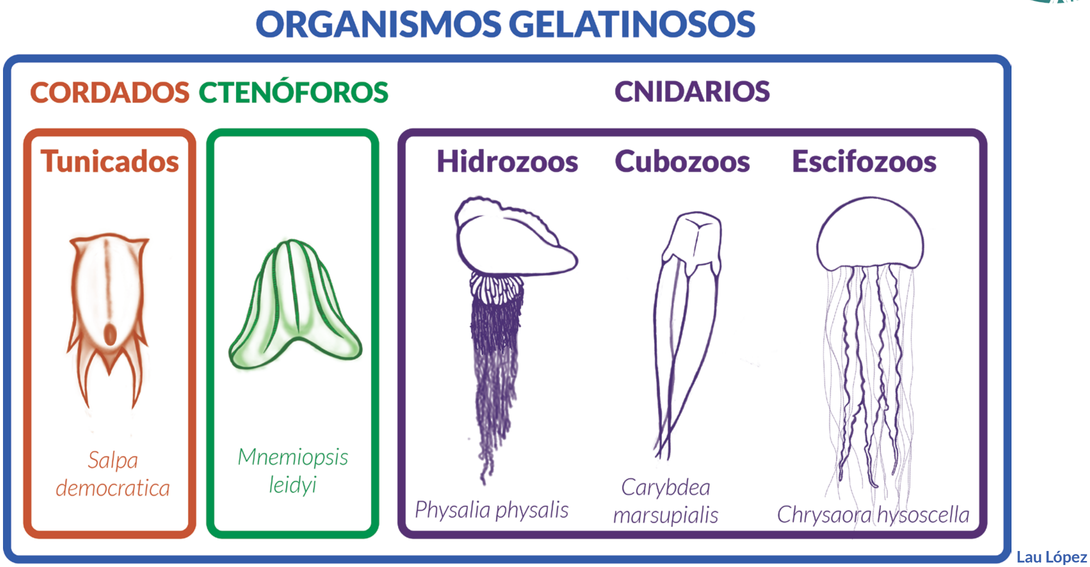

El filum Chordata está representado por el subfilum Tunicata que incluye a las salpas, organismos gelatinosos sin células urticantes. El filum Ctenophora que incluye a los ctenóforos que presentan diversas formas y se caracterizan por la presencia de 8 bandas longitudinales de cilios. El filum Cnidaria, que incluye a los corales, las anémonas, las gorgonias y al grupo más numeroso de medusas clasificadas en cuatro Clases: Staurozoa, Hydrozoa, Cubozoa y Scyphozoa, que son conocidas como las "verdaderas medusas".

Ciclo de vida de las medusas

Las medusas presentan diferentes ciclos de vida dependiendo de la especie. El que se presenta en esta guía es el más común para las medusas de la costa española, donde se alternan diferentes formas de vida y modelos reproductivos.

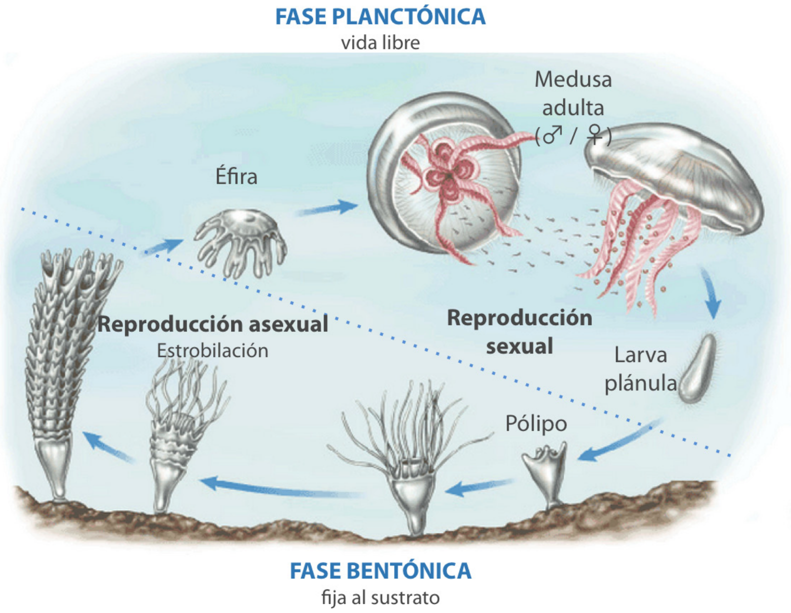

Los huevos fertilizados de medusas adultas se transforman en larvas llamadas plánulas, que nadan hasta adherirse a un sustrato, dando lugar a un pólipo e iniciando la fase bentónica.

El pólipo crece, se desarrolla y cuando las condiciones ambientales son óptimas, el pólipo se reproduce de forma asexual mediante el proceso de estrobilación, segmentando su cuerpo y dando lugar a múltiples pequeñas éfiras. Las éfiras, que nadan libres por la columna de agua, dan el inicio a la fase pelágica.

Las éfiras crecen y se desarrollan formando las medusas adultas que se reproducen sexualmente con fecundación interna o externa, produciendo huevos fértiles que iniciarán el ciclo nuevamente.

Anatomía de las medusas

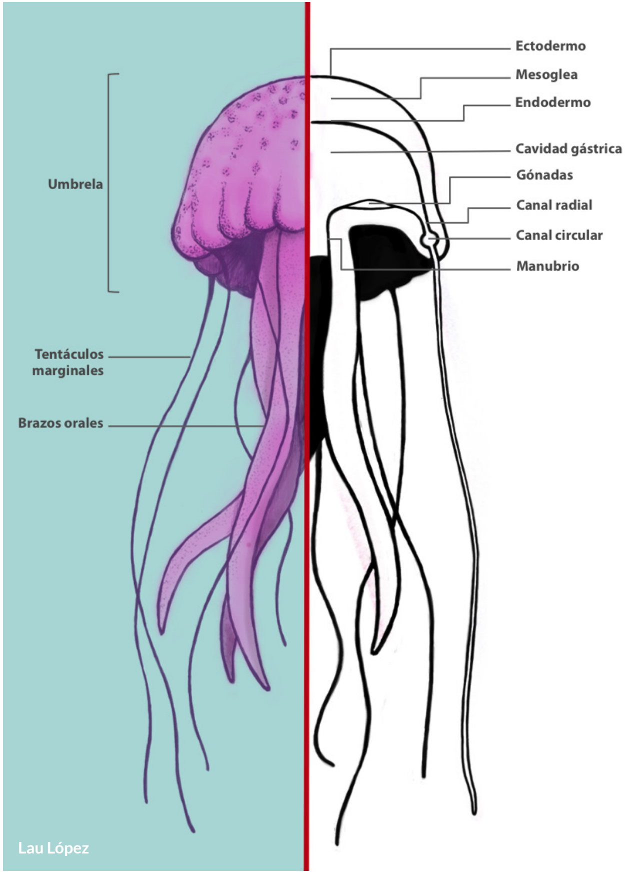

Las medusas están compuestas principalmente de agua (95%). Las medusas están formadas de una variedad de tejidos, con una capa exterior o ectoderma y una interior o endoderma, separadas por una capa gelatinosa llamada mesoglea.

Su organización o morfología general es bastante simple. Presentan un saco en el extremo, en forma de campana llamado umbrela. En el interior se encuentran las estructuras encargadas de la digestión (cavidad gástrica) y la reproducción (gónadas). En el centro de la cara cóncava de la umbrela hay un orificio que hace la función de boca y de orificio excretor. Alrededor de este orificio se encuentran los brazos orales, que son tentáculos gruesos agrupados que forman una especie de tubo llamado manubrio. En algunas especies, desde el borde de la umbrela nacen los tentáculos marginales. El número y longitud varía según la especie.

¿Por qué pican las medusas?

Las medusas tienen células urticantes especializadas llamadas cnidocitos. Se utilizan principalmente para alimentarse y defenderse. Están distribuidas por todo el cuerpo del animal pero más concentradas a lo largo de los tentáculos.

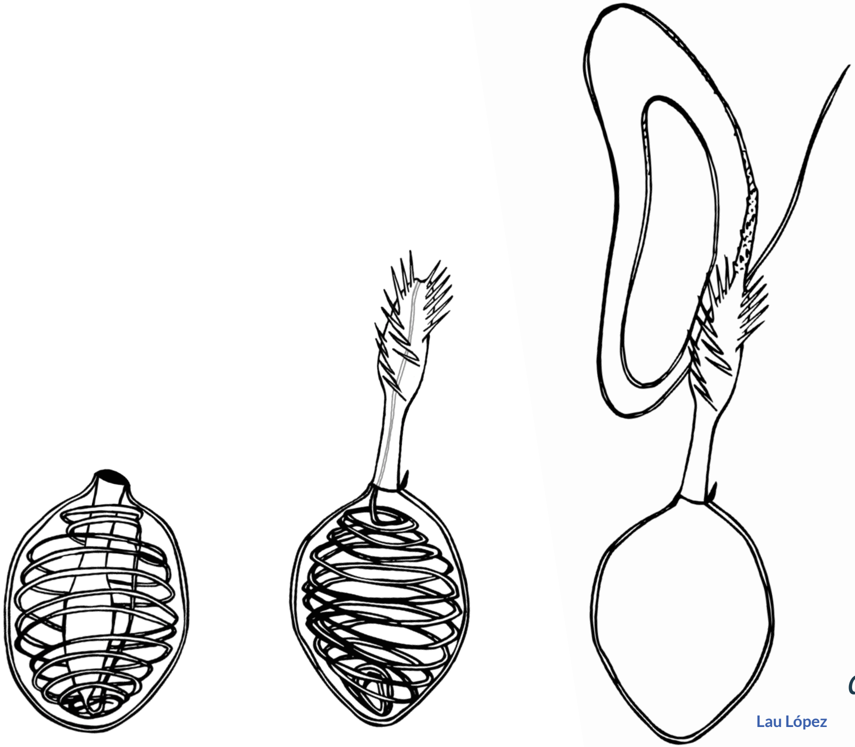

Lau López

Cada cnidocito contiene una cápsula con un filamento enrollado, casi siempre con espinas, y con toxinas. Cuando se activa, la cápsula se abre y el filamento se dispara penetrando al animal y vaciando el veneno contenido en la cápsula. El mecanismo se lleva a cabo en menos de una millonésima de segundo, lo que genera un impacto en el punto de penetración de más de 70 toneladas por centímetro cuadrado. Está considerado uno de los procesos de exocitosis más rápidos del reino animal.

Capacidad urticante y peligrosidad

La capacidad tóxica de una medusa persiste después de su muerte durante bastante tiempo. El grado de toxicidad del veneno varía dependiendo de la especie. La capacidad urticante de cada especie se clasifica en esta guía en 4 categorías:

CAPACIDAD TÓXICA

MUY URTICANTE

Especie con alta capacidad urticante con efectos importantes sobre la salud humana. Se recomienda evitar todo contacto con esta medusa.

URTICANTE

Especie con mediana capacidad urticante que podría producir efectos importantes sobre la salud humana. Se recomienda evitar todo contacto con esta medusa.

POCO URTICANTE

Especie con baja capacidad urticante con muy poco o casi ningún efecto sobre la salud humana. Aún así, se recomienda no tocarla ya que podría haber algún tipo de reacción dérmica post-contacto.

NO URTICANTE

Especie sin células urticantes. Completamente inofensiva para la salud humana.

LA MAR DE MEDUSAS

Recomendaciones generales para el tratamiento de picaduras producidas por medusas

NO aplicar amoníaco
NO aplicar alcohol
NO frotar
NO aplicar vendajes a presión
NO aplicar vinagre
(excepto en casos específicos)

Protocolo de actuación inmediata en caso de picadura de medusas

¿Reconoces la especie que te ha picado?

NO

Aplica el protocolo general.

Se utiliza en caso de picadura de
Pelagia noctiluca,
Rhizostoma pulmo,
Chrysaora hysoscella,
de otras especies urticantes
y en caso de especie no identificada

PROTOCOLO GENERAL

1

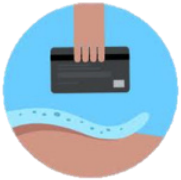

Eliminar los restos de tentáculos y/o fragmentos de medusas

2

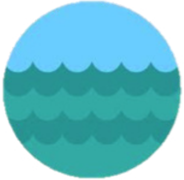

Lavar con agua de mar

3

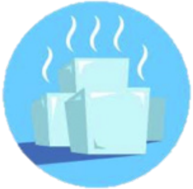

Aplicar frío por 15’ (en lapsos de 3’ +2’ de descanso)

4

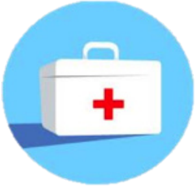

Si el dolor persiste, consultar al médico o profesional de salud

SÍ

Utiliza la guía para saber qué protocolo aplicar.

***En caso de duda, aplicar el protocolo general.**

Características de la picadura de las diferentes especies de medusas a modo general

Pelagia noctiluca

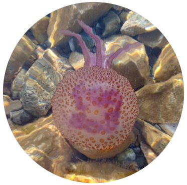

Su picadura produce una sensación de picor, dolor intenso, inflamación y enrojecimiento de la piel. La picadura produce urticaria y edema, además de vesículas, pápulas o costras que pueden aparecer y permanecer. Otros síntomas, aunque son poco comunes, pueden ser náuseas, vómitos, calambres musculares y dificultad respiratoria.

Rhizostoma pulmo

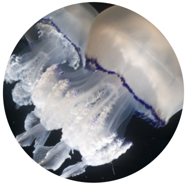

Su picadura puede ser dolorosa aunque en general produce un ligero escozor y una irritación intensa. El contacto con esta medusa o con fragmentos de ella puede producir cuadros dermatológicos en algunas personas, pero generalmente no son graves. Esta especie libera un moco que contiene células urticantes y aún cuando no haya un contacto directo, sí que podría haber una reacción dermatológica al estar en contacto con el agua.

Physalia physalis

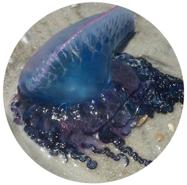

El veneno de las células urticantes de esta especie tiene propiedades neurotóxicas, citotóxicas y cardiotóxicas. El contacto puede producir escozor y dolor intenso y en algunos casos reacciones sistémicas. En la zona de contacto suele aparecer una línea de pápulas blancas y un margen rojo. Entre los efectos sistémicos que podrían observarse se incluyen temblores, diarrea, vómitos y convulsiones.

Características de la picadura de las diferentes especies de medusas a modo general

Chrysaora hysoscella

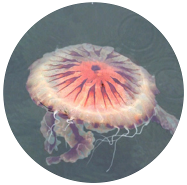

Su picadura generalmente produce un dolor fuerte que escuece y picor los primeros 20 minutos después del contacto y que generalmente desaparece las primeras dos horas. También podrían aparecer eritematoso y edemas durante las 48 horas siguientes al contacto. En muy pocos casos se han reportado síntomas más severos.

Carybdea marsupialis

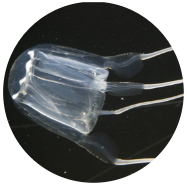

La picadura de esta especie es muy dolorosa, pero los efectos tienen corta duración. Generalmente aparecen unas pápulas rojizas en la piel y en algunos casos excepcionales se pueden observar calambres musculares, vómitos, cansancio y ansiedad.

Olindias muelleri

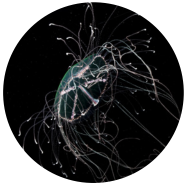

Después de la picadura de esta especie aparecen unas líneas rojizas en zig-zag en la piel que son muy características. El dolor es intenso e instantáneo.

Índice de las espécies de la guía

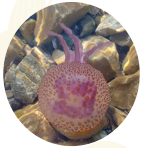

Pelagia noctiluca
página 13

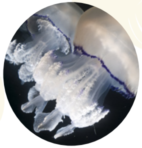

Rhizostoma pulmo
página 15

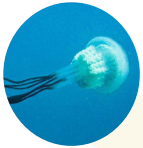

Rhizostoma luteum
página 17

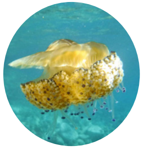

Cotylorhiza tuberculata
página 18

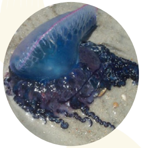

Physalia physalis
página 19

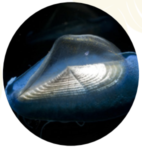

Velella velella
página 21

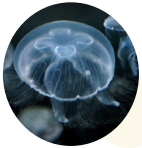

Aurelia spp.
página 23

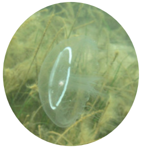

Discomedusa lobata
página 24

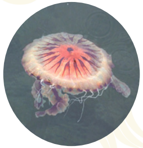

Chrysaora hysoscella
página 25

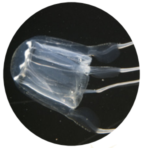

Carybdea marsupialis
página 27

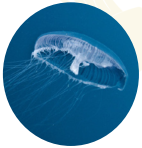

Aequorea forskalea
página 29

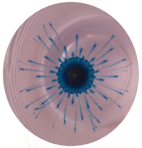

Porpita porpita
página 30

Índice de las espécies de la guía

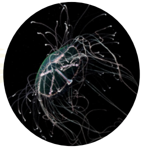

Olindias muelleri
página 31

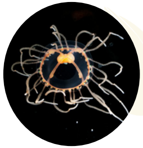

Gonionemus vertens
página 32

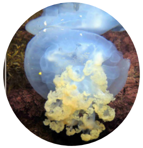

Catostylus tagi
página 33

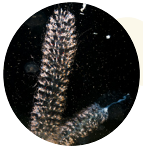

Sifonóforos
página 34

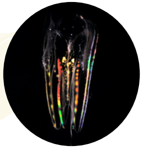

Salpas
página 35

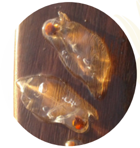

Leucothea multicornis
página 36

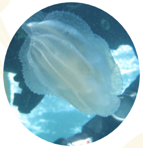

Beroe ovata
página 37

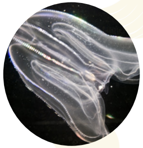

Mnemiopsis leidyi
página 38

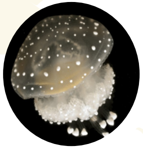

Phyllorhiza punctata
página 39

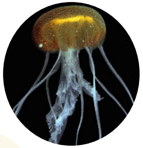

Otras medusas del Mediterráneo
página 40

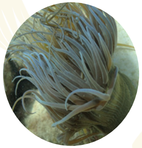

Otros Cnidarios del Mediterráneo
página 44

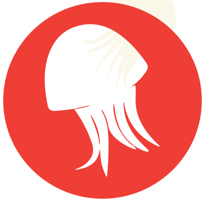

MUY URTICANTE

Umbrela hasta 25 cm
4 brazos orales
8 tentáculos marginales

CNIDARIO
Escifozoo

Pelagia noctiluca

Medusa luminiscente o clavel

Descripción

Color rosa rojizo. Los juveniles pueden ser de color marrón claro a oscuro. El diámetro de la umbrela puede alcanzar hasta 25 cm. La umbrela es semiesférica con 4 brazos orales y 8 tentáculos marginales (que extendidos alcanzan hasta 2 m de longitud). La superficie de la umbrela está cubierta de verrugas marrones.
Es una especie oceánica y su presencia en la costa depende de las condiciones ambientales y climatológicas.
Es una especie bioluminiscente.

EN CASO DE PICADURA SEGUIR LAS RECOMENDACIONES DEL PROTOCOLO GENERAL

1

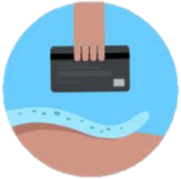

Eliminar los restos de tentáculos y/o fragmentos de medusas

2

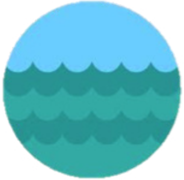

Lavar con agua de mar

3

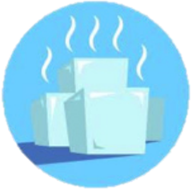

Aplicar frío por 15’ (en lapsos de 3’ +2’ de descanso)

4

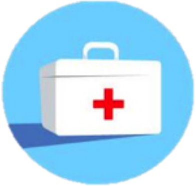

Si el dolor persiste, consultar al médico o profesional de salud

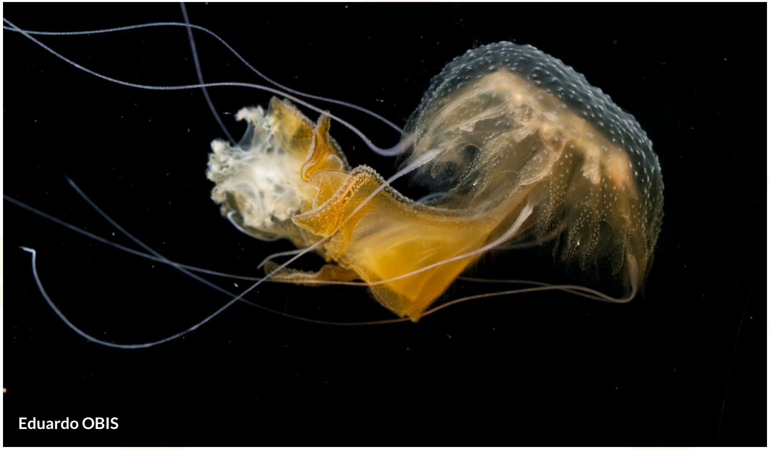

Eduardo OBIS

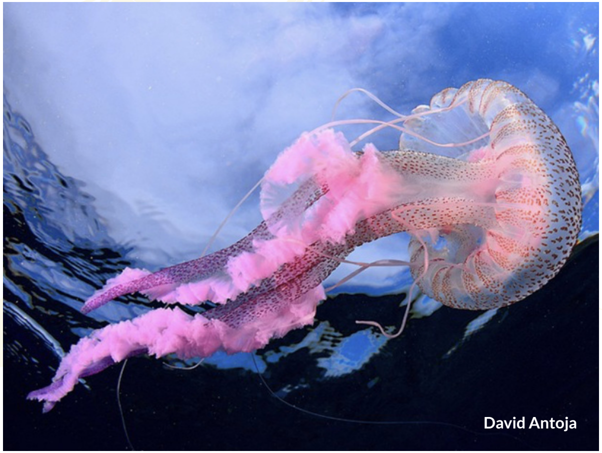

David Antoja

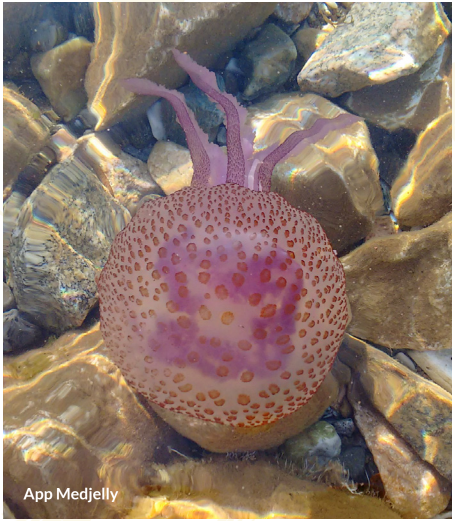

App Medjelly

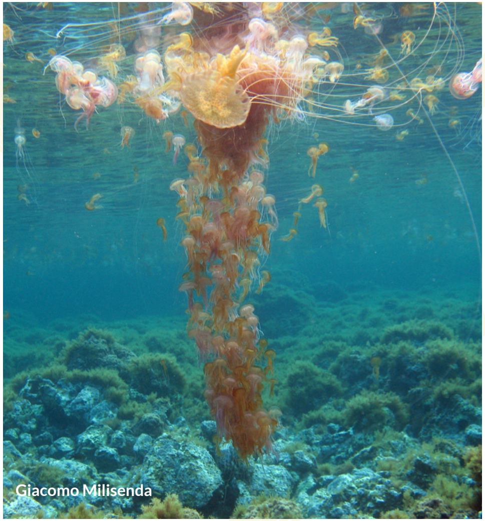

Giacomo Milisenda

Su ciclo de vida es completamente pelágico. Los huevos fertilizados se transforman en plánulas que darán lugar directamente a las pequeñas éfiras sin la presencia de pólipo ni fase bentónica.

Rhizostoma pulmo

Acalefo azul

URTICANTE

Umbrela hasta 40 cm
8 brazos orales
Sin tentáculos marginales

CNIDARIO
Escifozoo

EN CASO DE PICADURA SEGUIR LAS RECOMENDACIONES DEL PROTOCOLO GENERAL
página 8

Descripción

El diámetro de la umbrela puede alcanzar hasta 40 cm.
La umbrela es acampanada de color blanco azulado y con un ribete violeta. No tiene tentáculos marginales pero si 8 brazos orales gruesos de color blanco azulado, fusionamos y sin ramificaciones.
Es una de las medusas más grandes de la costa mediterránea española.

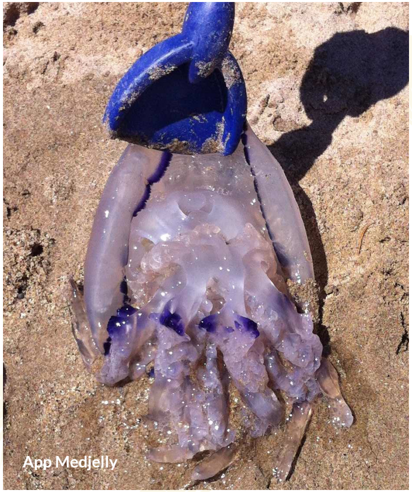

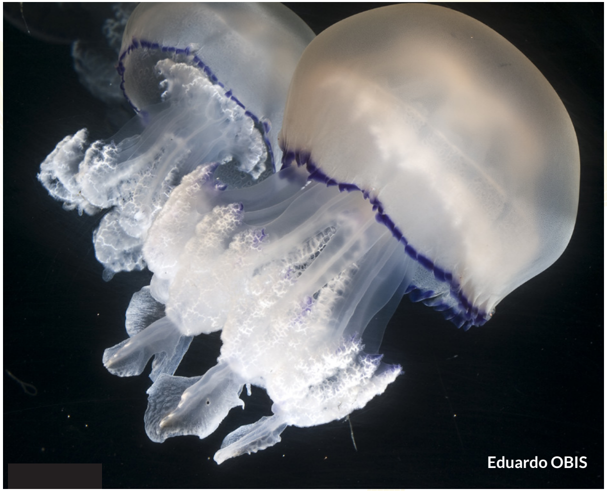

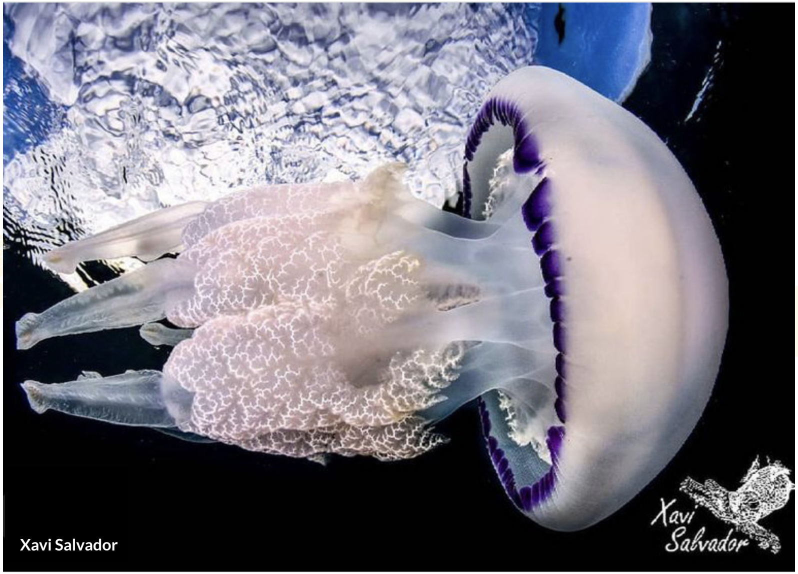

Xavi Salvador

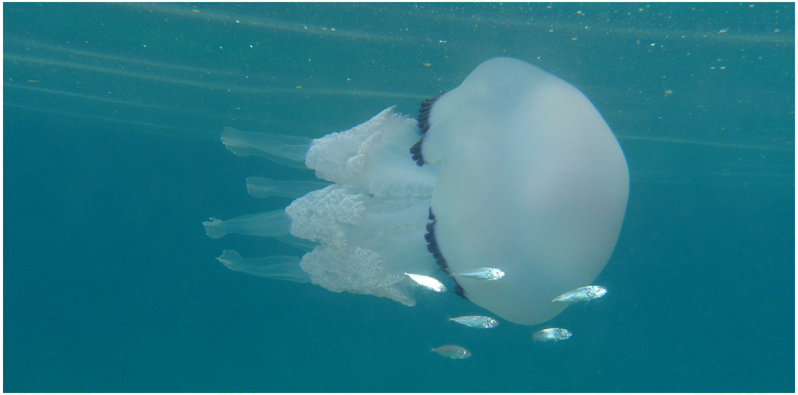

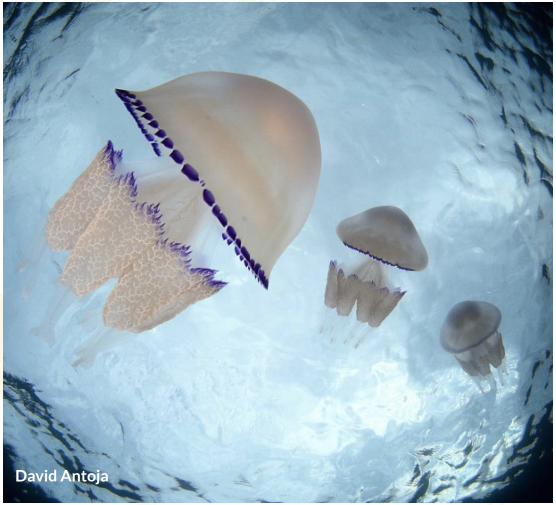
David Antoja

Las medusas pequeñas son visibles durante la primavera y los adultos de mayor tamaño son más evidentes durante el verano y al comienzo del otoño en la costa mediterránea española. Es una especie costera, que se distribuye a lo largo de la columna de agua. Suelen estar acompañadas por cangrejos y peces juveniles que la utilizan como refugio.

Rhizostoma luteum

URTICANTE

Umbrela hasta 70cm
8 brazos orales
Sin tentáculos marginales

CNIDARIO
Escifozoo

EN CASO DE PICADURA SEGUIR LAS RECOMENDACIONES DEL PROTOCOLO GENERAL página 8

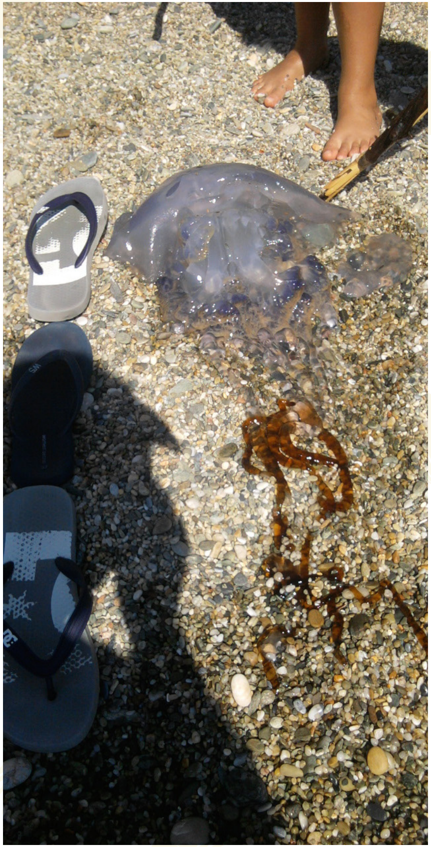

Descripción

Umbrela acampanada de color blanco azulado que mide hasta 70 cm de diámetro, con ausencia del ribete violeta característico de la especie R. pulmo. Sin tentáculos marginales y con 8 brazos orales de coloración gris-negra en la parte distal, que pueden medir hasta 2 m de longitud.
Se puede observar sola o formando enjambres. Fue descrita por primera vez en 1827 en el Mediterráneo, y a partir del 2012 se comenzó a observar de forma más frecuente.

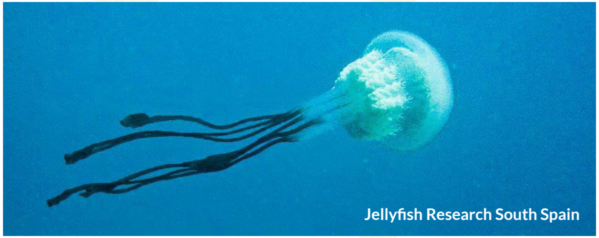

Jellyfish Research South Spain

Cotylorhiza tuberculata

Medusa huevo frito

Descripción

El diámetro de la umbrela puede alcanzar los 35 cm. La umbrela tiene forma aplanada, es marrón-amarillenta y tiene una gran protuberancia central de color naranja más oscuro. No tiene tentáculos marginales pero sí consta de 8 brazos orales con 3 apéndices en el extremo en forma de botón y de color blanco o azul. Es una especie costera, endémica del Mediterráneo y con preferencia de aguas más cálidas. Los adultos son más abundantes a finales del verano y a principios del otoño en la costa mediterránea española. Suelen estar acompañadas de peces juveniles que la utilizan como refugio.

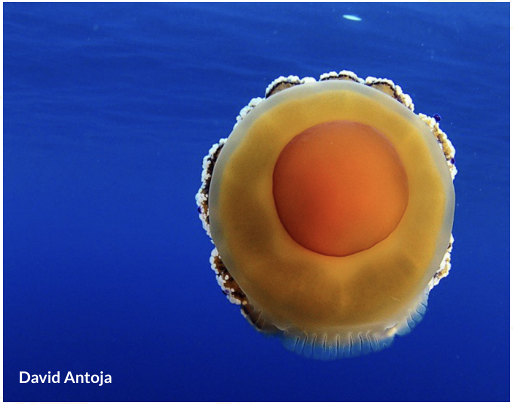

David Antoja

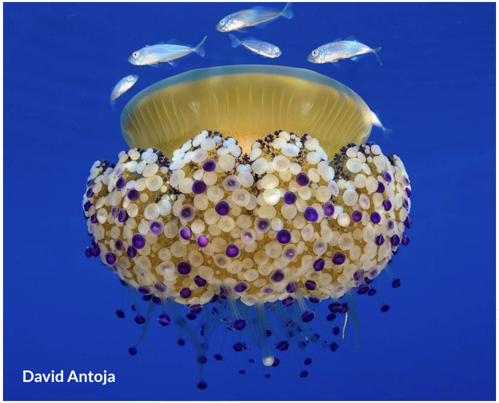

David Antoja

POCO URTICANTE

Umbrela hasta 35 cm
8 brazos orales
Sin tentáculos marginales

CNIDARIO
Escifozoo

EN CASO DE PICADURA SEGUIR LAS RECOMENDACIONES DEL PROTOCOLO GENERAL
página 8

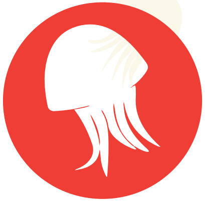

MUY URTICANTE

**Parte flotante
hasta 30 cm de largo
y 10 cm de ancho
Tentáculos 20 m**

CNIDARIO
Hidrozoo
Sifonóforo

Physalia physalis

Carabela portuguesa

Descripción

Es un organismo colonial del orden de los sifonóforos. Está compuesto por una cámara llena de gas, violeta y translúcida coronada por una vela. La parte flotante puede alcanzar hasta 30 cm de largo y 10 cm de ancho. La parte sumergida está formada por tentáculos azules finos y largos que extendido pueden llegar a medir 20 m. Es nativa del océano Atlántico pero ocasionalmente (en primavera) puede observarse en algunas zonas del Mediterráneo.

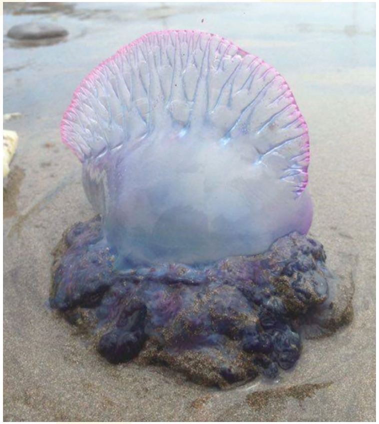

EN CASO DE PICADURA SEGUIR LAS RECOMENDACIONES DEL PROTOCOLO ESPECÍFICO 1

1

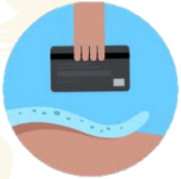

Eliminar los restos de tentáculos y/o fragmentos de medusas

2

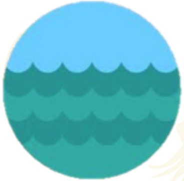

Lavar con agua de mar

3

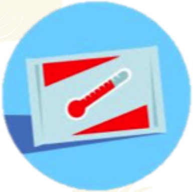

Aplicar Hot packs o inmersión en agua caliente (40º o 45º) durante 15’

4

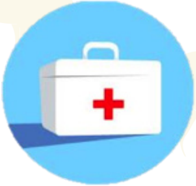

Si el dolor persiste, consultar al médico o profesional de salud

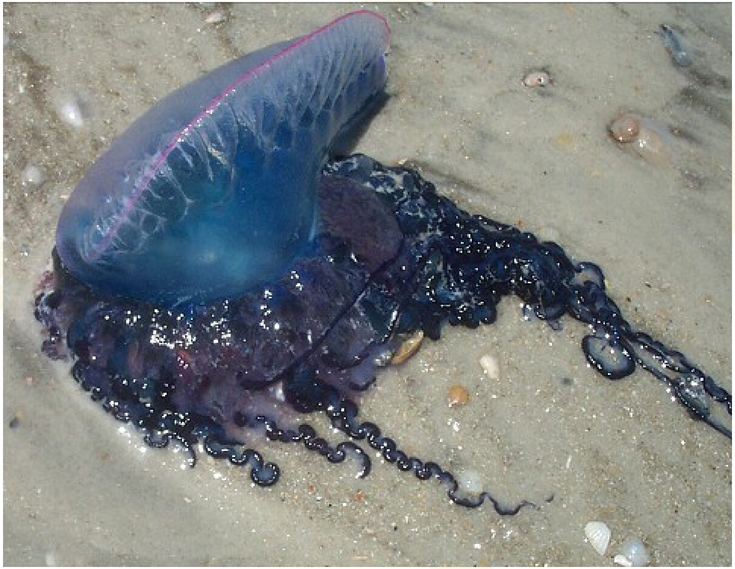

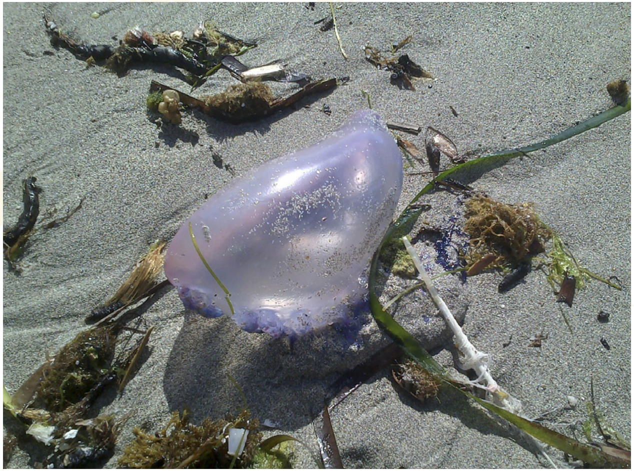

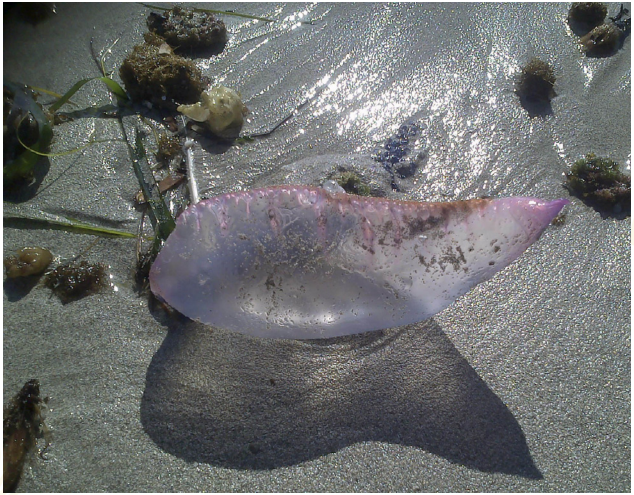

Cada conjunto de organismos que forman la colonia está especializado en diversas tareas que aseguran la supervivencia de la especie.

El neumatóforo proporciona la flotabilidad . Los dactilozoides se encargan de la defensa y de atrapar el alimento que es digerido por los gastrozoides. Los gonozoides están especializados en la reproducción.

Velella velella

Barquitas De San Pedro o vela púrpura

POCO URTICANTE

Disco ovalado
de hasta 8 cm
Vela triangular
Colonia de pólipos
especializados

CNIDARIO
Hidrozoo

EN CASO DE PICADURA SEGUIR LAS RECOMENDACIONES DEL PROTOCOLO GENERAL
página 8

Descripción

Es una hidromedusa y la fase de su ciclo de vida que normalmente observamos es una colonia de pólipos flotantes. Está compuesta por un disco ovalado azul que puede alcanzar hasta 8 cm de diámetro y una vela triangular perpendicular al disco. Bajo el velo del disco se encuentran los pólipos especializados, encargados de producir pequeñas medusas que se reproducen sexualmente para formar nuevas colonias flotantes.

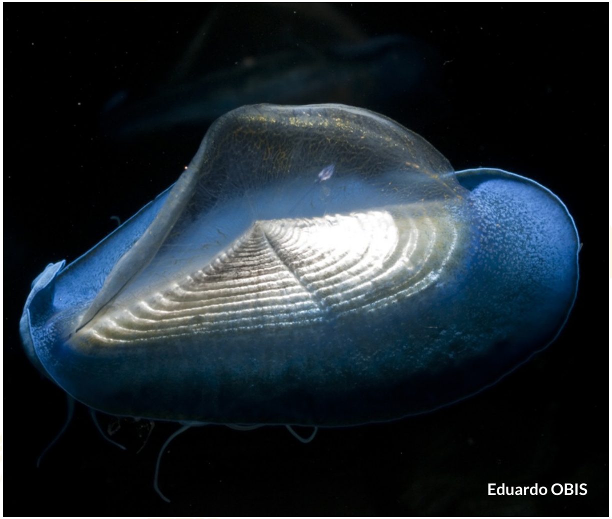
Eduardo OBIS

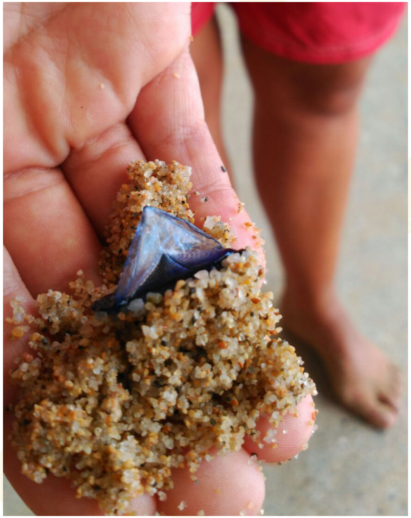

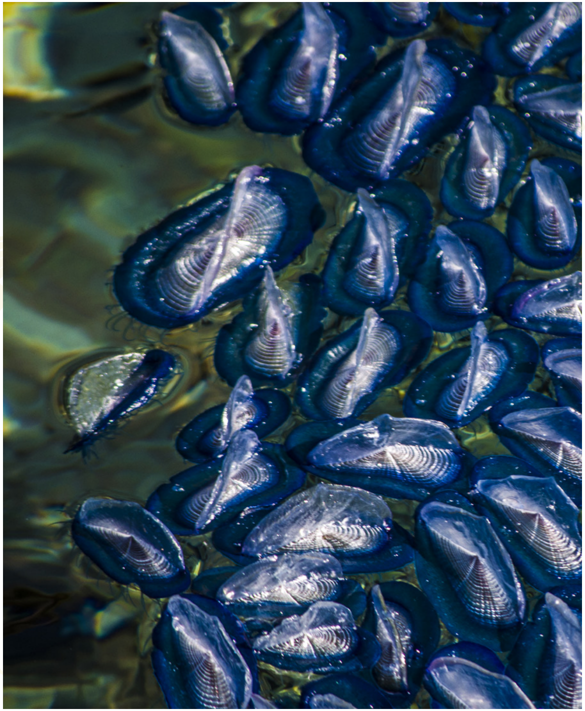

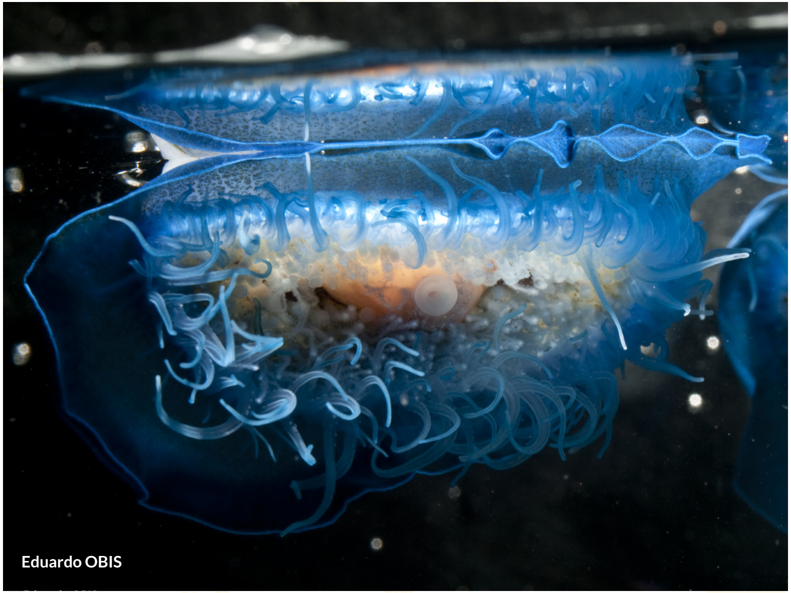

Eduardo OBIS

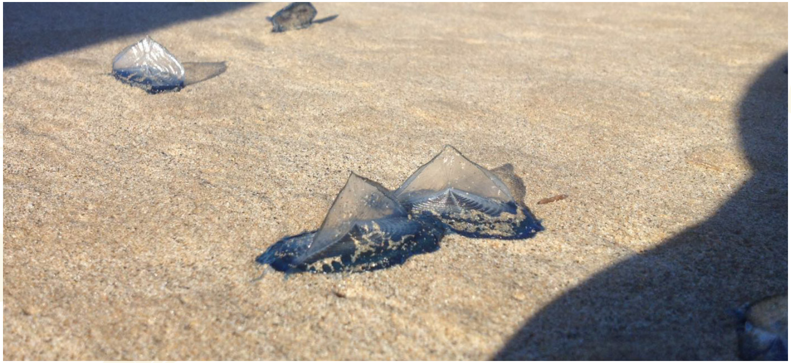

Es una especie frecuente en el Mediterráneo y en el Atlántico, especialmente hacia finales del invierno y durante la primavera, llegando a formar grandes enjambres que alcanzan la costa.

POCO URTICANTE

Umbrela hasta 25 cm
Centenares de tentáculos marginales
4 brazos orales
Gónadas en forma de herradura

CNIDARIO
Escifozoo

EN CASO DE PICADURA SEGUIR LAS RECOMENDACIONES DEL PROTOCOLO GENERAL
página 8

Aurelia spp.

Medusa luna o común

Descripción

El diámetro de la umbrela puede medir hasta 25 cm. La umbrela es translúcida con forma aplanada. Tiene 4 brazos orales largos y centenares de tentáculos marginales cortos y finos. En la umbrela se observan 4 órganos reproductores (gónadas) en forma de herradura y de color blanquecino amarillento o ligeramente rosa.
Es una especie cosmopolita. Es una especie costera que se puede encontrar incluso en estuarios y puertos.

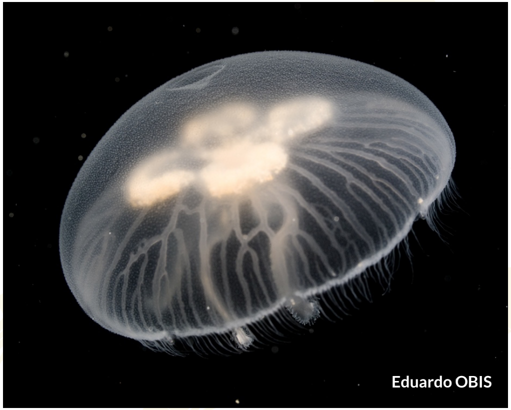
Eduardo OBIS

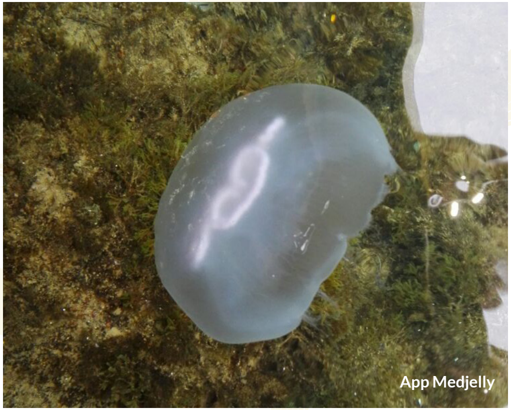
App Medjelly

Discomedusa lobata

Descripción

El diámetro de la umbrela puede medir hasta unos 10-15 cm. La umbrela es aplanada y translúcida con 4 gónadas visibles de color blanquecino. Tiene 4 brazos orales y 48 tentáculos marginales muy finos. En el MEditerráneo era considerada una especie rara, pero desde el año 2013 es bastante abundante en ciertas zonas de la costa de Cataluña. Esta especie se suele confundir con Aurelia spp. La característica que permite diferenciarlas fácilmente es la forma de las gónadas. En D. lobata, ocupan todo el perímetro de la umbrela formando un único cordón, mientras que en Aurelia spp. presentan forma de herradura en el centro de la umbrela.

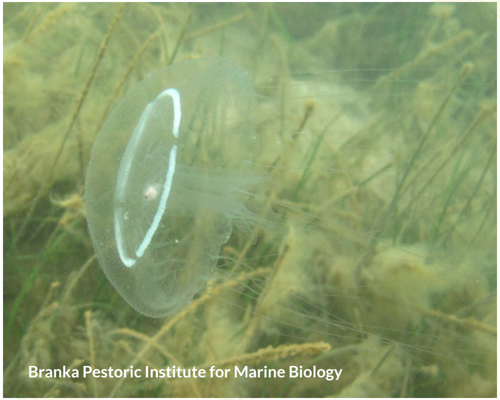
Branka Pestoric Institute for Marine Biology

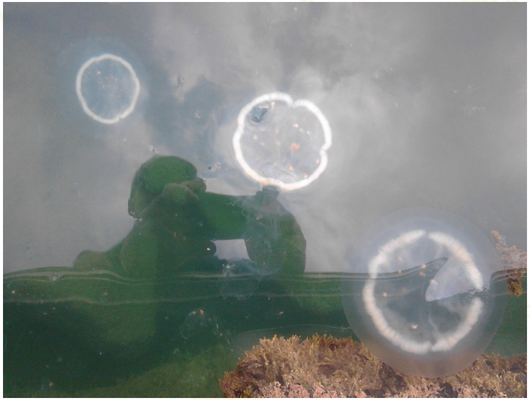

POCO URTICANTE

Umbrela hasta 15 cm
48 tentáculos marginales
4 brazos orales
Gónadas en forma de cordón

CNIDARIO
Escifozoo

EN CASO DE PICADURA SEGUIR LAS RECOMENDACIONES DEL PROTOCOLO GENERAL
página 8

Chrysaora hysoscella

Acalefo radiante o medusa compás

MUY URTICANTE

Umbrela hasta 30 cm
16 bandas marrones
4 brazos orales
24 tentáculos marginales

CNIDARIO
Escifozoo

Descripción

El diámetro de la umbrela puede medir hasta 30 cm. La umbrela es semiesférica y aplanada de color blanco amarillento, con 16 bandas marrones en forma de V en la superficie. Tiene 4 brazos orales largos de hasta 1 m de longitud y 24 tentáculos marginales largos y finos.

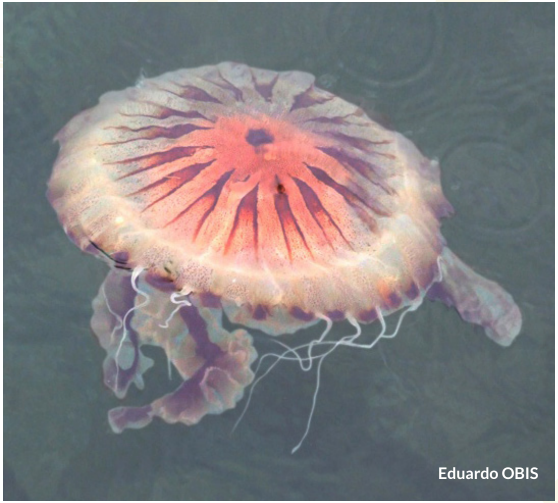
Eduardo OBIS

EN CASO DE PICADURA SEGUIR LAS RECOMENDACIONES DEL PROTOCOLO GENERAL

1

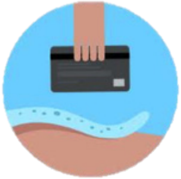
Eliminar los restos de tentáculos y/o fragmentos de medusas

2

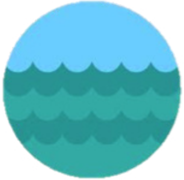
Lavar con agua de mar

3

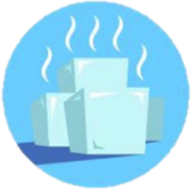
Aplicar frío por 15’ (en lapsos de 3’ +2’ de descanso)

4

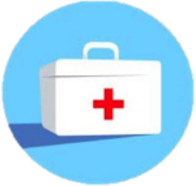
Si el dolor persiste, consultar al médico o profesional de salud

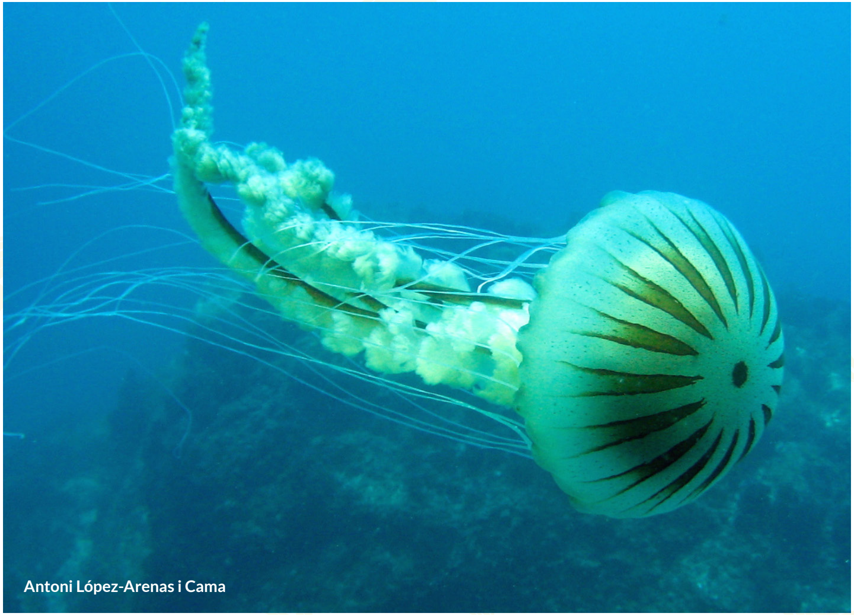
Antoni López-Arenas i Cama

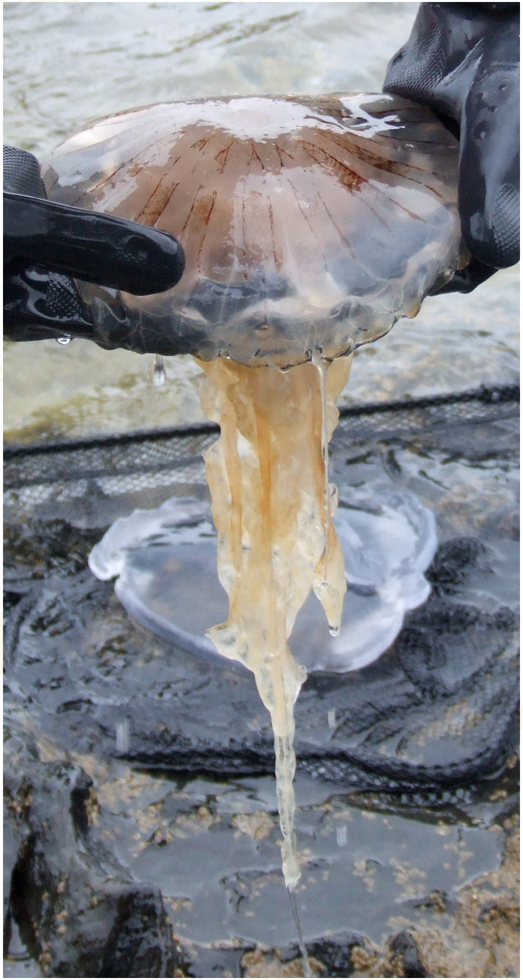

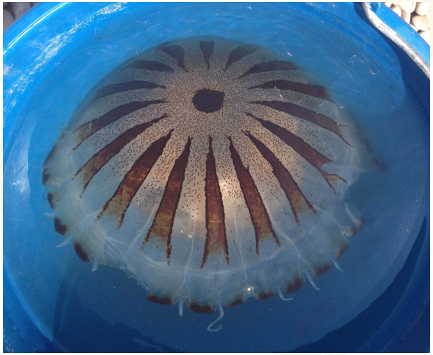

Puede ser bastante abundante en algunas áreas del Mediterráneo español aunque raramente forma enjambres. Normalmente está presente durante la primavera. Suelen estar acompañadas de peces juveniles.

Carybdea marsupialis

Cubomedusa o avispa de mar

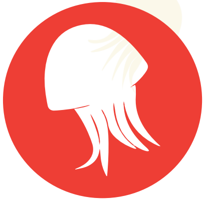

MUY URTICANTE

Umbrela hasta 5 cm
Forma de cubo
4 tentáculos
Sin brazos orales

CNIDARIO
Cubozoo

Descripción

El diámetro de la umbrela puede alcanzar hasta 5 cm. La umbrela tiene forma de cubo, es translúcida y de una coloración blanca azulada. Tiene 4 tentáculos largos que nacen de cada extremo de la umbrela. C. marsupialis es la única especie de cubomedusa conocida del Mediterráneo, pero su veneno no es letal.

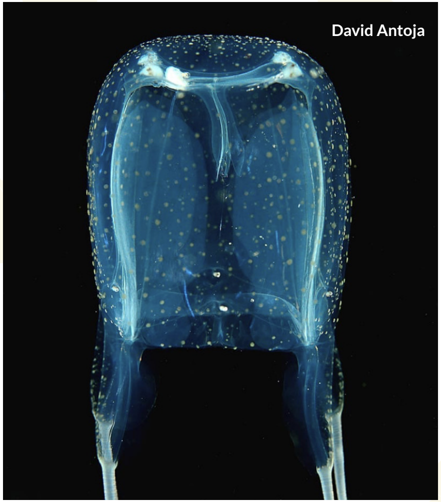

EN CASO DE PICADURA SEGUIR LAS RECOMENDACIONES DEL PROTOCOLO ESPECÍFICO 2

1

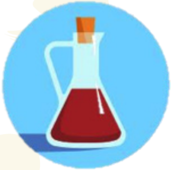

Lavar con vinagre comercial

2

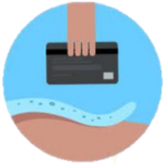

Eliminar los restos de tentáculos y/o fragmentos de medusas

3

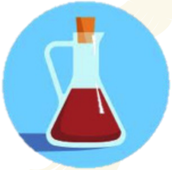

Lavar nuevamente con vinagre comercial

4

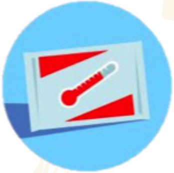

Aplicar Hot packs o inmersión en agua caliente (40° o 45°) durante 15’

5

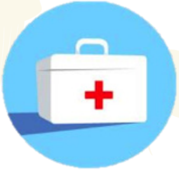

Si el dolor persiste, consultar al médico o profesional de salud

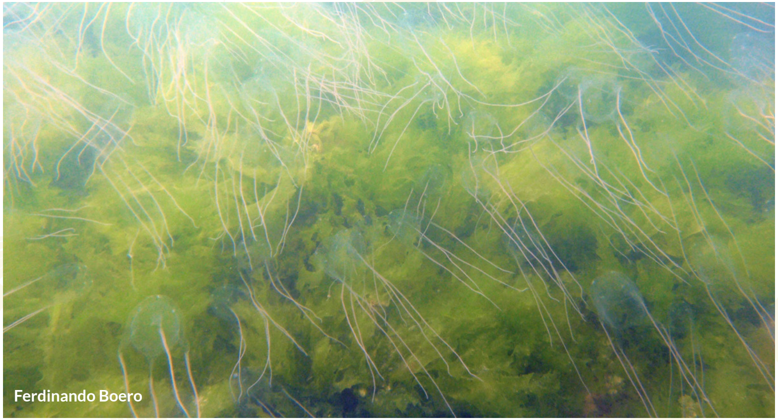

Ferdinando Boero

Se suele encontrar en aguas poco profundas. El ambiente preferido de esta especie es el sustrato arenoso, y se localiza en el fondo durante el día, desplazándose hacia la superficie durante la noche.
Es muy abundante en ciertas áreas de la costa mediterránea española.

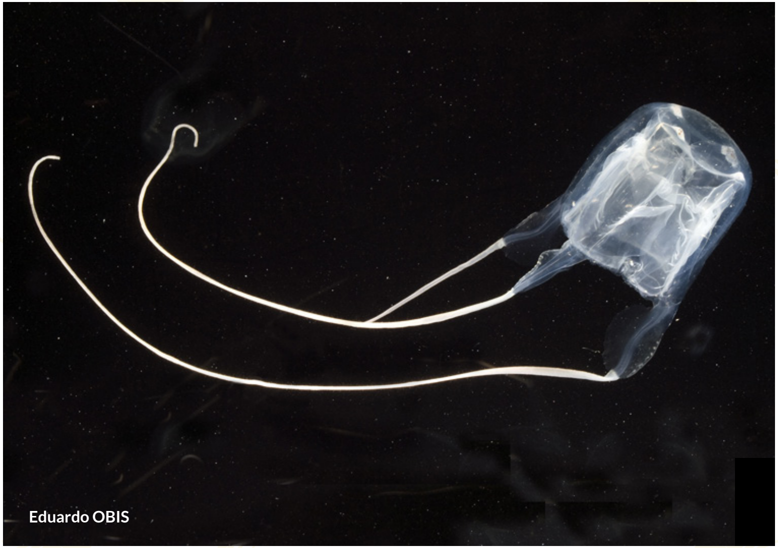

Eduardo OBIS

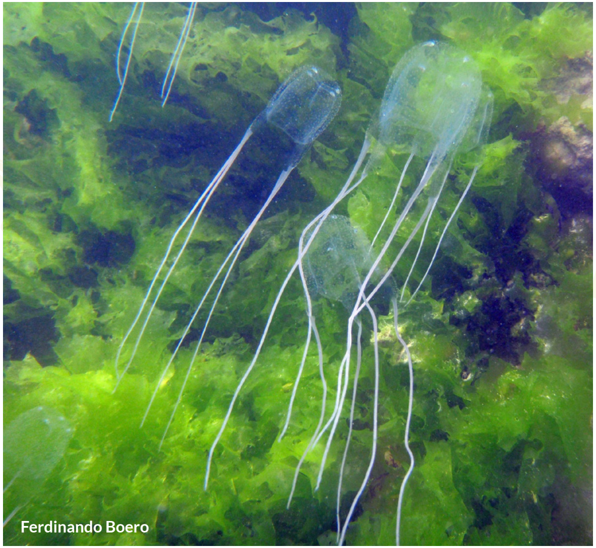

Ferdinando Boero

Aequorea forskalea

Medusa aequorea

POCO URTICANTE

Umbrela hasta 40 cm
Numerosos tentáculos
Sin brazos orales
Canales radiales de color azul

CNIDARIO
Hidrozoo

EN CASO DE PICADURA SEGUIR LAS RECOMENDACIONES DEL PROTOCOLO GENERAL
página 8

Descripción

Es una hidromedusa con la umbrela aplanada y más densa en el centro. El diámetro de la umbrela puede alcanza hasta 40 cm. La umbrela es translúcida con canales radiales de color azul. Tiene numerosos tentáculos finos y no presenta brazos orales.
Esta especie habita desde aguas templadas a tropicales, en áreas costeras y litorales, apareciendo incluso de forma ocasional en mar abierto. Es una especie frecuente en el Mediterráneo español, siendo más común durante la primavera y formando grandes enjambres ocasionales.

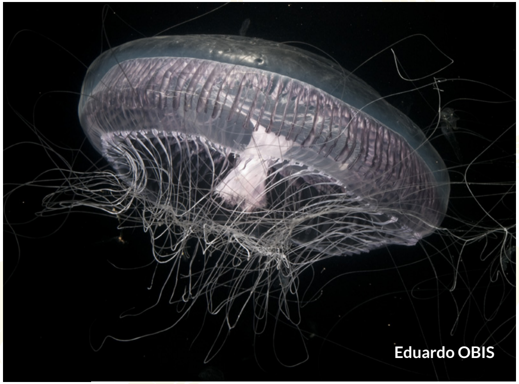

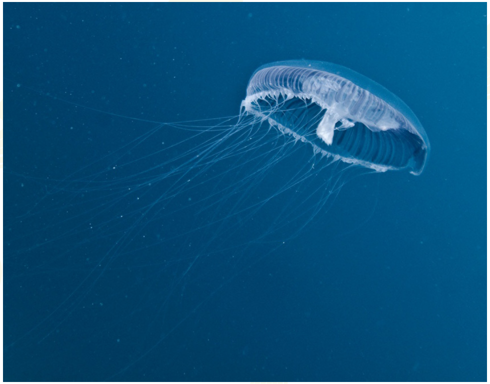

Porpita porpita

Botón azul

Descripción

Es una hidromedusa de la cual normalmente observamos la fase de su ciclo de vida que corresponde a una colonia flotante de pólipos. El diámetro del disco puede alcanzar 5 cm y es de color azul intenso. Se compone de dos partes: un flotador duro, marrón dorado y lleno de gas en el centro, y de una colonia de hidroides de color azul intenso, morados o amarillos, que parecen tentáculos. Es un organismo colonial que vive en la superficie de mar abierto, pero puede encontrarse en zonas costera en grandes cantidades. Es ocasional en el mar Mediterráneo.

POCO URTICANTE

Disco hasta 5 cm
Flotador duro
Colonia de hidroides
Color azul intenso

CNIDARIO
Hidrozoo

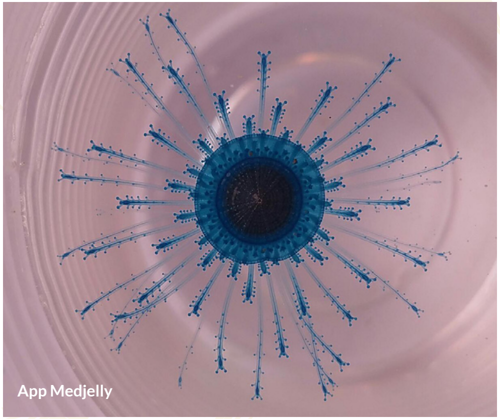

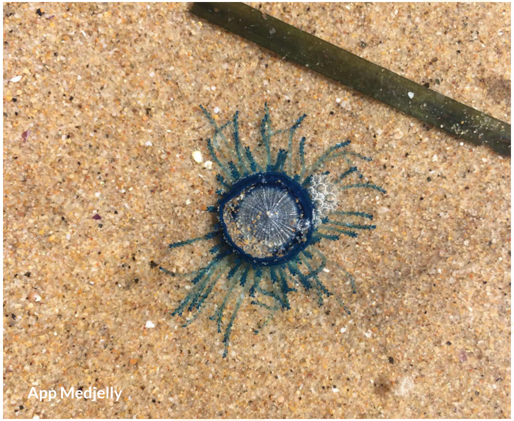

EN CASO DE PICADURA SEGUIR LAS RECOMENDACIONES DEL PROTOCOLO GENERAL
página 8

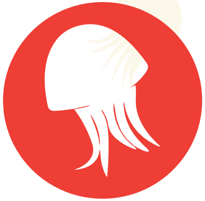

Olindias muelleri

(Olindias phosphorica anteriormente)

Medusa cruz

MUY URTICANTE

Umbrela hasta 8 cm
Muchos tentáculos cortos
Sin brazos orales
Gónadas en cruz

CNIDARIO
Hidrozoo

Descripción

Hidromedusa de umbrela abombada, translúcida, de hasta 8 cm de diámetro, rodeada de muchos tentáculos cortos de color morado. Presenta 4 gónadas en forma de cruz de color granate. Tiene pigmentación fluorescente visible durante la noche. Localizada en zonas específicas de la costa catalana.

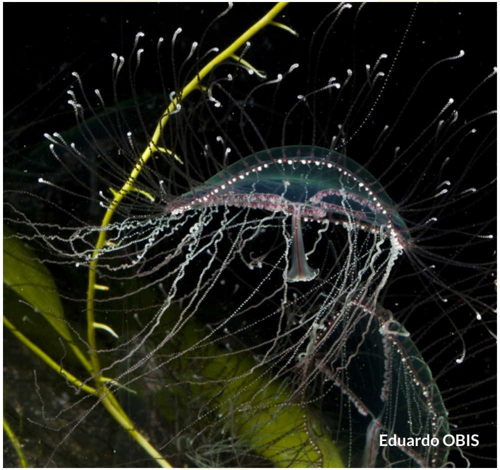

EN CASO DE PICADURA SEGUIR LAS RECOMENDACIONES DEL PROTOCOLO GENERAL

1

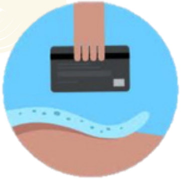

Eliminar los restos de tentáculos y/o fragmentos de medusas

2

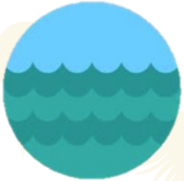

Lavar con agua de mar

3

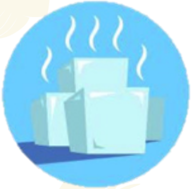

Aplicar frío por 15’ (en lapsos de 3’ +2’ de descanso)

4

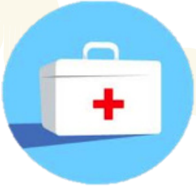

Si el dolor persiste, consultar al médico o profesional de salud

Gonionemus vertens

Medusa de rayas naranjas

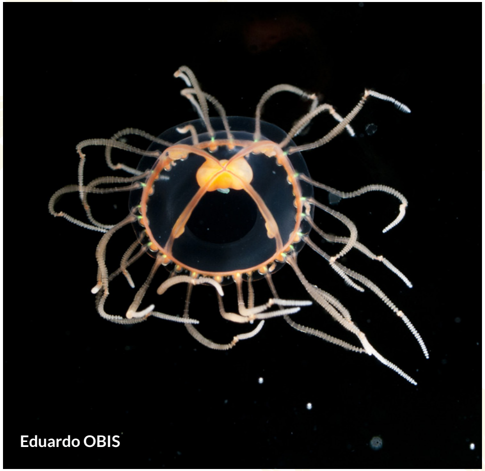

Descripción

Hidromedusa con una umbrela que puede medir hasta 4cm de diámetro, es translúcida con las gónadas de color naranja. La rodean numerosos tentáculos, hasta 90, con ventosas en los extremos, por lo que es frecuente encontrarla adherida a algas marinas. Es una especie introducida en el Mediterráneo.

MUY URTICANTE

Umbrela hasta 4 cm
Muchos tentáculos cortos
Sin brazos orales
Gónadas naranja

CNIDARIO
Hidrozoo

1

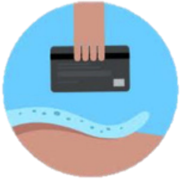

Eliminar los restos de tentáculos y/o fragmentos de medusas

2

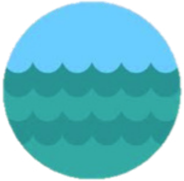

Lavar con agua de mar

3

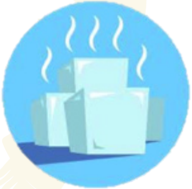

Aplicar frío por 15’ (en lapsos de 3’ +2’ de descanso)

4

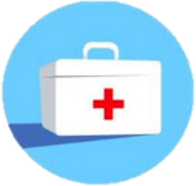

Si el dolor persiste, consultar al médico o profesional de salud

EN CASO DE PICADURA SEGUIR LAS RECOMENDACIONES DEL PROTOCOLO GENERAL

Catostylus tagi

URTICANTE

Umbrela hasta 30 cm
Sin tentáculos marginales
8 brazos orales

CNIDARIO
Escifozoo

EN CASO DE PICADURA SEGUIR LAS RECOMENDACIONES DEL PROTOCOLO GENERAL **página 8**

Descripción

Tiene una umbrela semiesférica de coloración amarillenta, blanca azulada o a veces marrón, con 8 brazos orales cónicos en forma de coliflor. El diámetro de la umbrela puede alcanzar hasta 30 cm. Es una especie que no forma enjambres.
Se encuentra en zonas poco profundas y aguas costeras, generalmente en bahías y puertos. Es una especie introducida en el Mediterráneo. Entró por el Estrecho de Gibraltar.

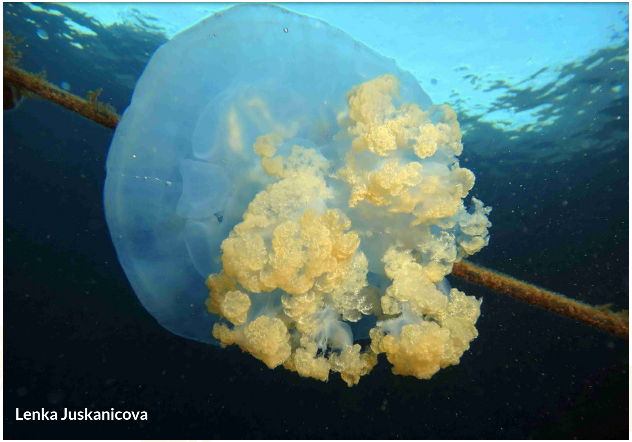
Lenka Juskanicova

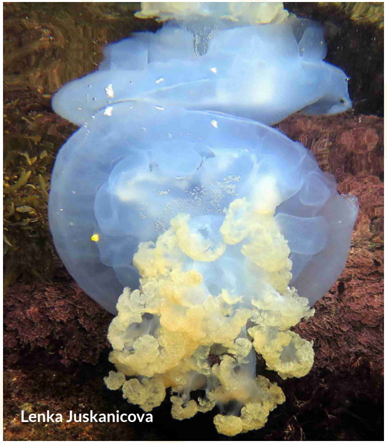
Lenka Juskanicova

Sifonóforos

Descripción

Los sifonóforos forman parte del holoplancton (completan todo su ciclo vital en la columna de agua, sin fase bentónica).
Son organismos coloniales. Están presentes durante todo el año pero en la primavera presentan sus máximas abundancias, aún cuando es fácil encontrarlos también en el verano.
Los más comunes en la costa Mediterránea española son Abylopsis tetragona, Chelophyes appendiculata y Muggiaea atlantica.

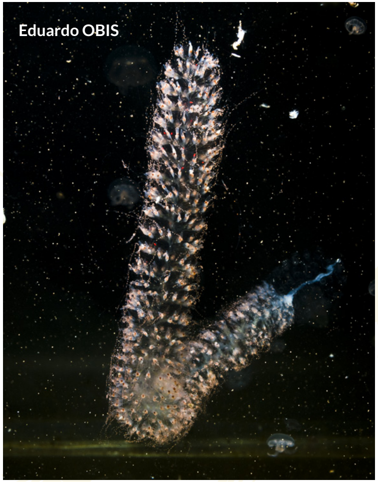

La fotografía muestra una especie del género Forskalia. Corresponde a una colonia de centenares de pólipos cuya estructura es cónica con un tamaño de 1 a 3 m de largo. En estas colonias hay individuos con funciones especializadas en la flotabilidad y el movimiento de la colonia, otros en la nutrición de todo el organismo y otros en la reproducción. Como todos los Cnidarios, poseen células urticantes y su capacidad urticante varía según la especie.

CAPACIDAD URTICANT E SEGÚN LA ESPÉCIE

CNIDARIO
Hidrozoo
Sifonóforo

EN CASO DE PICADURA SEGUIR LAS RECOMENDACIONES DEL PROTOCOLO GENERAL
página 8

Salpas

NO URTICANTE

CHORDATA
Tunicado

Descripción

Tunicados pelágicos gelatinosos. Son organismos translúcidos con un cuerpo en forma de barril. Se distingue claramente en un extremo del cuerpo, el estómago como un botón azul (en el caso de Thalia democratica) o de color marrón rojizo (en el caso de diversas especies como por ejemplo, en Salpa fusiformis).
En la costa catalana suelen aparecer durante la primavera y el otoño.

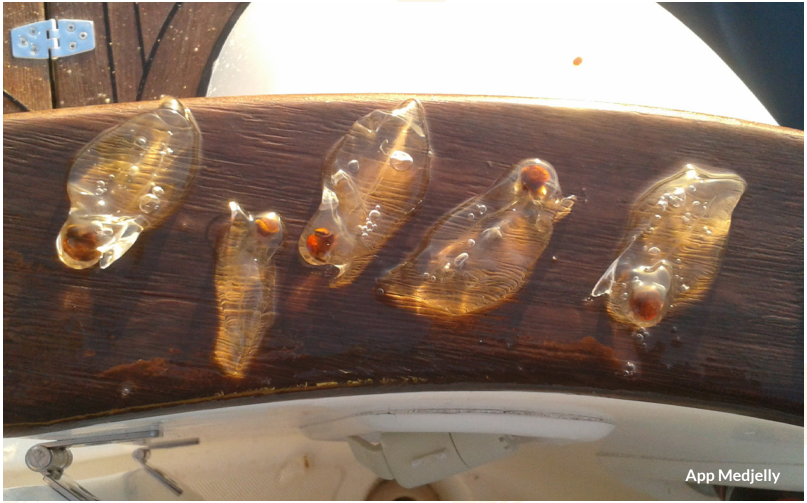
App Medjelly

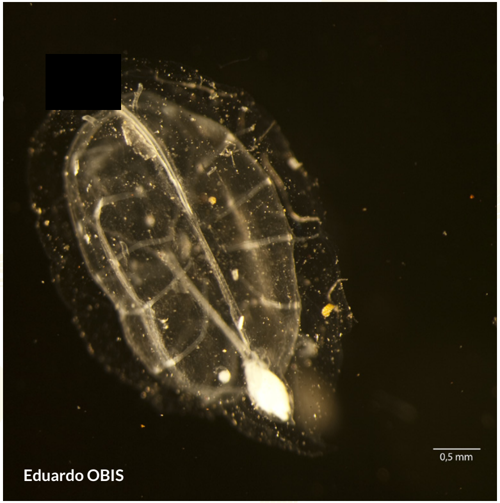

Eduardo OBIS

Leucothea multicornis

Descripción

Es un ctenóforo y no posee células urticantes. Tiene un cuerpo translúcido, lateralmente comprimido, que puede alcanzar hasta 20 cm de longitud. La superficie del cuerpo está cubierta por numerosas papilas retráctiles e hileras de cilios brillantes. Presente dos lóbulos subdivididos en dos que salen de la mitad de la boca y dos tentáculos largos y finos, uno a cada costado del cuerpo.

Suelen ser abundantes en la época de primavera, coincidiendo con la proliferación de muchos organismos del plancton gelatinoso.

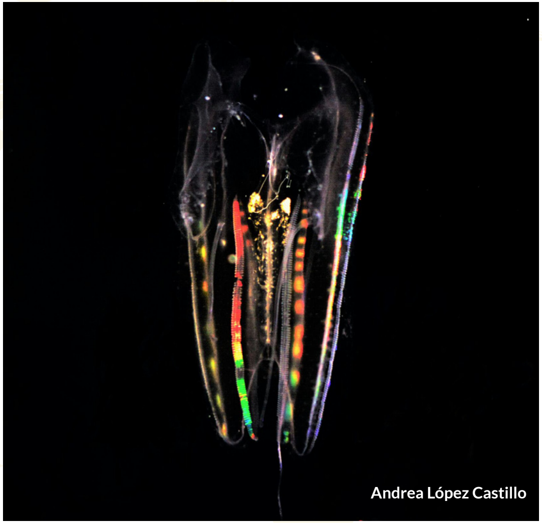

Andrea López Castillo

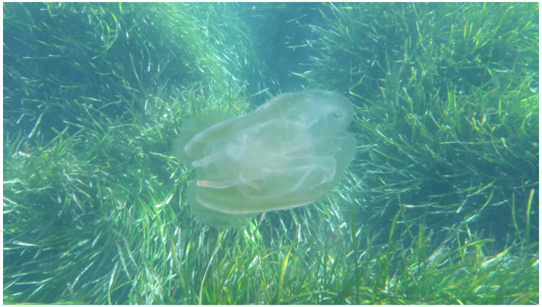

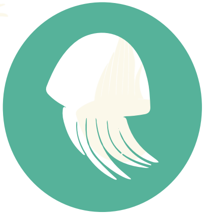

NO URTICANTE

Cuerpo hasta 20 cm
Fusiforme
Lóbulos laterales

CTENÓFORO

Beroe ovata

NO URTICANTE

Longitud hasta 30 cm
Forma ovalada
8 hileras de cilios

CTENÓFORO

Beroe ovata

Globo de mar

Descripción

Es un ctenóforo, organismo gelatinoso que no posee células urticantes. Tiene una longitud que puede alcanzar hasta 30 cm. Cuerpo de forma ovalada o cilíndrica con una compresión lateral muy marcada.
Es translúcido, con una coloración azulada pálida o a veces rosa pálido. En la superficie presenta 8 hileras longitudinales de cilios y no posee tentáculos.
Es nativo del Atlántico occidental.

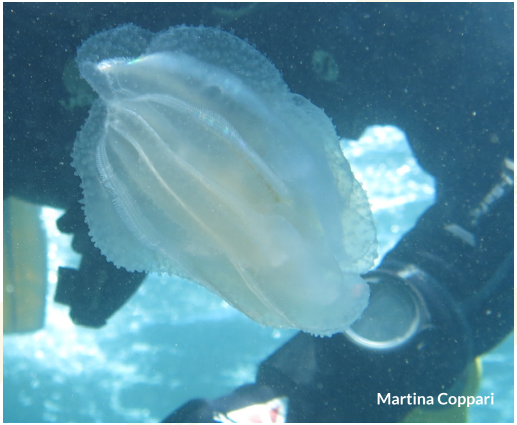

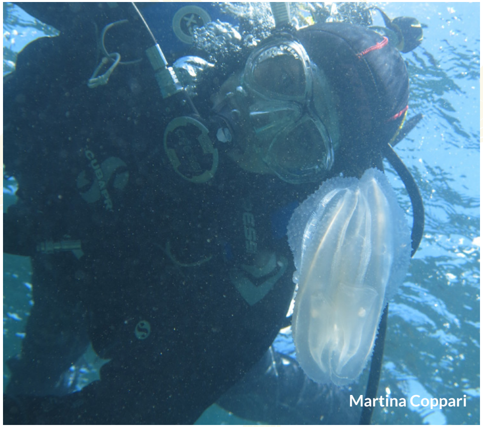

Mnemiopsis leidyi

Medusa bombilla o peine

Descripción

Ctenóforo nativo del Atlántico occicental. La longitud de su cuerpo puede alcanzar hasta 12 cm. Tiene forma ovalada y es translúcido. Los adultos poseen 8 hileras lineales de cilios en forma de peines y no tienen tentáculos. Es una especie invasora en el Mar MEditerráneo y en la costa catalan se encuentra restringido a zonas específicas. Esta especie es un ctenóforo y no tiene células urticantes, por lo que inofensivo para la salud humana, pero es muy nocivo para los ecosistemas invadidos.

NO URTICANTE

Longitud hasta 12 cm
Forma ovalada
8 hileras de cilios

CTENÓFORO

ESPÉCIE INVASORA EN EL MEDITERRÁNEO

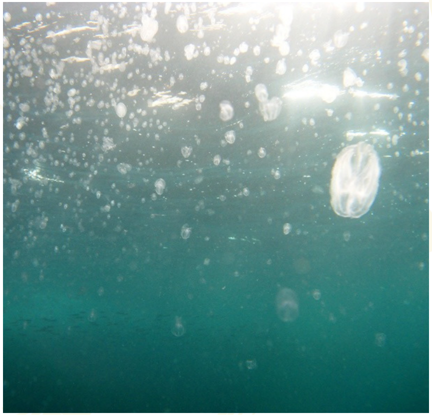

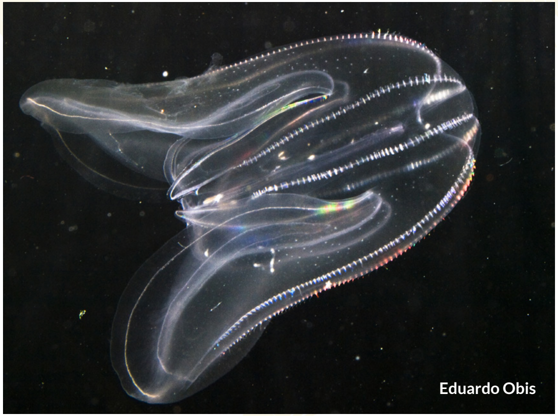

POCO URTICANTE

Umbrela hasta 70 cm
Sin tentáculos marginales
8 brazos orales

CNIDARIO
Escifozoo

ESPÉCIE INVASORA EN EL MEDITERRÁNEO

EN CASO DE PICADURA SEGUIR LAS RECOMENDACIONES DEL PROTOCOLO GENERAL página 8

Phyllorhiza punctata

Medusa de lunares blancos

Descripción

El diámetro de la umbrela puede alcanzar hasta 70 cm. Es semiesférica y tiene lunares blancos distribuidos uniformemente. No tiene tentáculos marginales pero posee 8 brazos orales gruesos, con 14 apéndices transparentes en el extremo. Puede presentar dos coloraciones, marrón verdosa oscura o blanco azulado.
Es nativa del Indo-Pacífico. Su primer registro en la costa catalana fue el año 2010 y se encuentra restringida a zonas específicas.

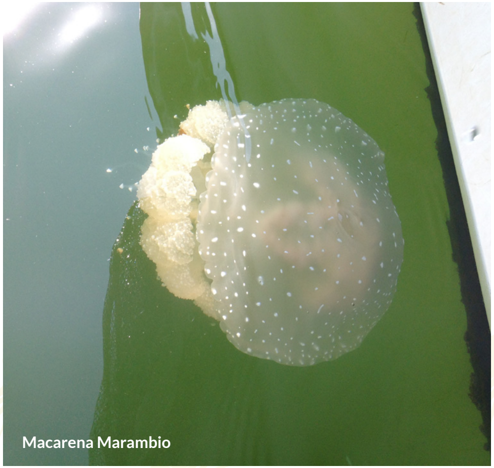
Macarena Marambio

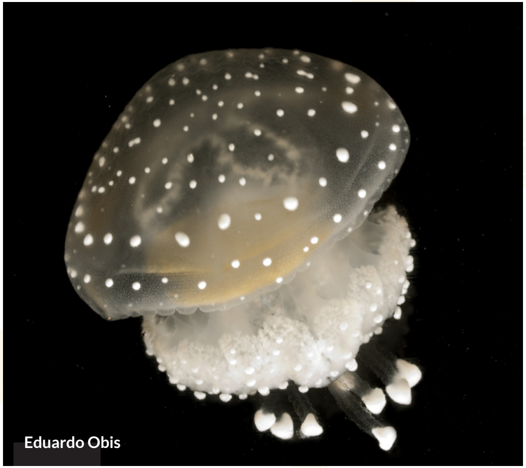
Eduardo Obis

Otras medusas del Mediterráneo

Mawia benovici

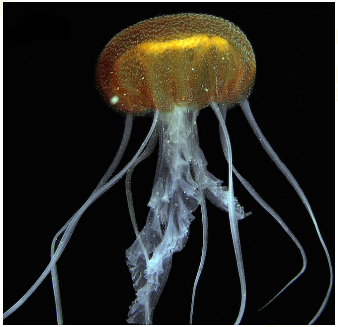

Umbrela semiesférica de color amarillo ocre con verrugas blanquecinas. 4 brazos orales y 8 tentáculos marginales.

Drymonema dalmatinum

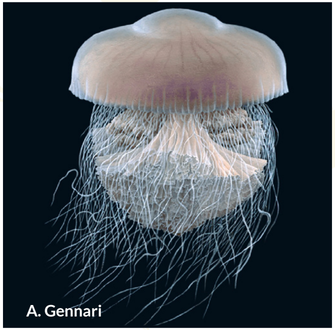

Umbrela aplanada y blanquecina, de hasta 1 m de diámetro. 4 brazos orales y numerosos tentáculos marginales.

Rhopilema nomadica

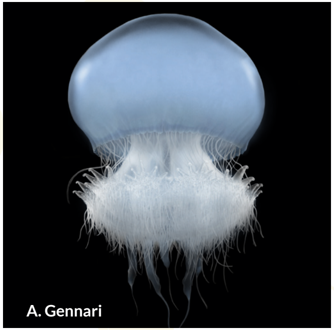

Especie introducida. Umbrela semiesférica, azul y translúcida de hasta 80 cm de diámetro. 8 brazos orales y numeroso tentáculos marginales.

MUY URTICANTE

CNIDARIO

Escifozoo

EN CASO DE PICADURA SEGUIR LAS RECOMENDACIONES DEL PROTOCOLO GENERAL

página 8

POCO URTICANTE

CNIDARIO
Escifozoo

Otras medusas del Mediterráneo

Cassiopea spp.

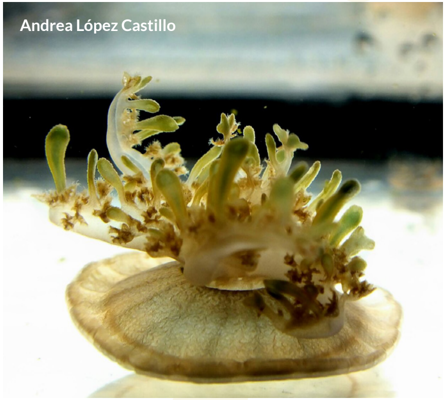

Conocida como medusa invertida. Umbrela aplanada de hasta 30 cm de diámetro, marrón amarillento y con lunares blancos. 8 brazos orales y sin tentáculos marginales.

Marivagia stellata

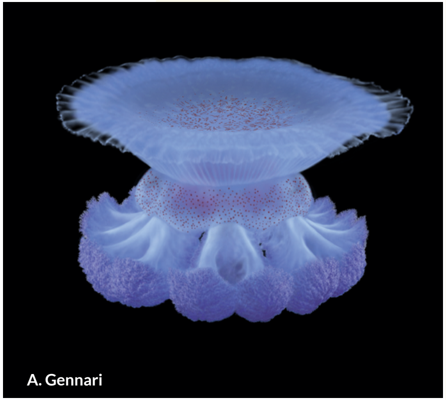

Especie introducida. Umbrela aplanada y translúcida blanca azulada de hasta 15 cm de diámetro. 8 brazos orales sin tentáculos marginales.

EN CASO DE PICADURA SEGUIR LAS RECOMENDACIONES DEL PROTOCOLO GENERAL
página 6

Otras medusas del Mediterráneo

Liriope tetraphylla

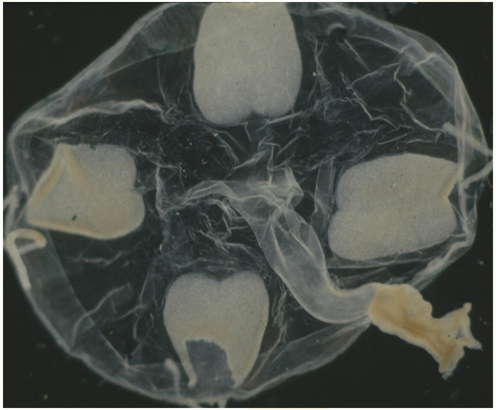

Hidromedusa muy común en el Mediterráneo. Umbrela semiesférica de hasta 30 mm de diámetro. Translúcida con 4 gónadas. 4 tentáculos largos.

Geryonia proboscidalis

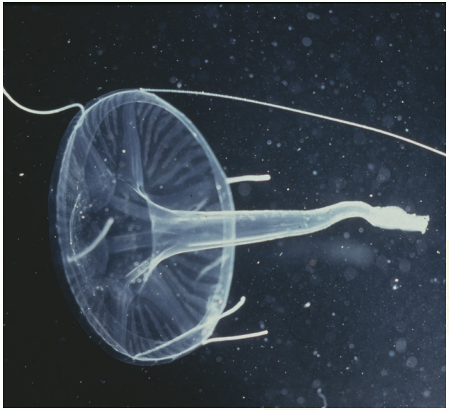

Hidromedusa con umbrela casi hemisférica con un diámetro de hasta 80 mm. Translúcida y con 6 gónadas. 12 tentáculos (6 largos y 6 cortos alternados).

MUY URTICANTE

CNIDARIO
Hidrozoo

EN CASO DE PICADURA SEGUIR LAS RECOMENDACIONES DEL PROTOCOLO GENERAL
página 6

MUY URTICANTE

CNIDARIO
Hidrozoo

EN CASO DE PICADURA SEGUIR LAS RECOMENDACIONES DEL PROTOCOLO GENERAL
página 8

Otras medusas del Mediterráneo

Neotima lucullana

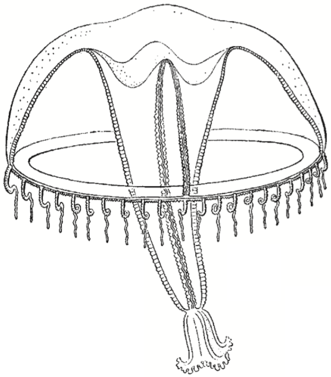

Hidromedusa con una umbrela más plana que hemisférica, de hasta 75 mm de diámetro. Translúcida con 70 tentáculos.

Solmissus albescens

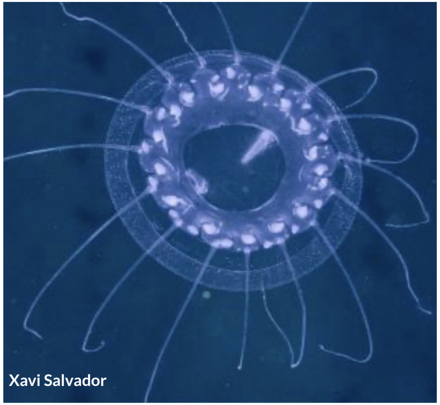

Xavi Salvador

Hidromedusa con una umbrela de hasta 50 mm de diámetro. Presenta 14-16 tentáculos casi tan largos como el diámetro de la umbrela.

Otros Cnidarios del Mediterráneo: Anémonas

Las anémonas no son organismos del zooplancton gelatinosos pero pertenecen al mismo filo que las medusas, Cnidaria. Son organismos marinos (aproximadamente unas 1000 especies) que carecen de esqueleto y presentan tentáculos largos con cnidocitos. Se incluyen en esta guía ya que se trata de organismos con capacidad urticante, aunque sólo pocas especies pueden provocar efecto perceptible en las personas, variando desde un picor muy débil hasta casos de gran intensidad que pueden incluir mucho dolor y cicatrices.

URTICANTE

CNIDARIO
Anthozoa

Anemonia viridis

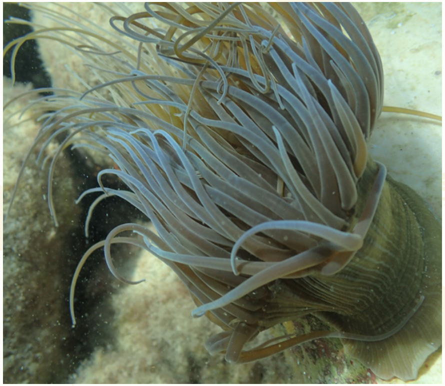

Conocida también como anémona común. Tiene tentáculos de coloración verde claro a gris verdoso violeta, con puntas a menudo violeta. Habita en costas rocosas someras y hasta 30 m de profundidad.

EN CASO DE PICADURA SEGUIR LAS RECOMENDACIONES DEL PROTOCOLO GENERAL
página 8

Precauciones ante la presencia de medusas en las playas

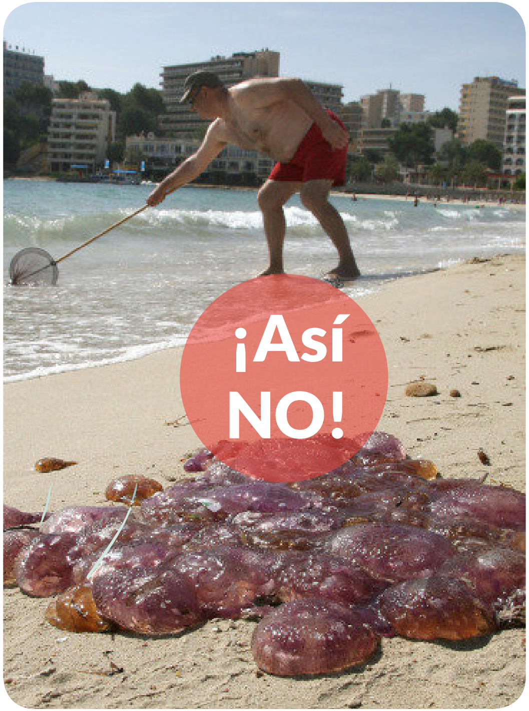

No retirar del agua las medusas porque con la manipulación pueden activarse las células urticantes. Los objetos que hayan tocado la medusa pueden quedar con restos de cnidocitos y provocar una picadura posterior.

No dejar las medusas en la arena porque sus restos siguen siendo urticantes por un tiempo.

Informar al personal de playa para que valore la situación.

Ante un encuentro con medusas en el agua, **nadar pausadamente y salir del agua sin movimientos bruscos.**

LA MAR DE MEDUSAS

Equipo del proyecto La Mar de Medusas

Coordinadora del área científica
Macarena Marambio

Coordinadora del área educativa
Janire Salazar

Coordinadora del área de difusión
Ainara Ballesteros

Responsable del proyecto
Josep-Maria Gili

Contáctanos a ***lamardemedusas@gmail.com***

@lamardemedusas

Autores: ***Macarena Marambio, Ainara Ballesteros, Laura López, Verónica Fuentes, Josep Maria Gili***
Diseño: ***Lau López*** *@laulopezarts*
